# Retinal artery occlusion and associated recurrent vascular risk with underlying etiologies

**DOI:** 10.1371/journal.pone.0177663

**Published:** 2017-06-01

**Authors:** Jeong-Ho Hong, Sung-Il Sohn, Jaehyuk Kwak, Joonsang Yoo, Seong Joon Ahn, Se Joon Woo, Cheolkyu Jung, Kyu Sun Yum, Hee-Joon Bae, Jun Young Chang, Jin-Heon Jung, Ji Sung Lee, Moon-Ku Han

**Affiliations:** 1 Department of Neurology, Keimyung University Dongsan Medical Center, Daegu, Korea; 2 Department of Ophthalmology, Hanyang University Hospital, Hanyang University College of Medicine, Seoul, Korea; 3 Department of Ophthalmology, Seoul National University Bundang Hospital, Seongnam, Korea; 4 Department of Radiology, Seoul National University Bundang Hospital, Seongnam, Korea; 5 Department of Neurology, Seoul National University Bundang Hospital, Seoul National University College of Medicine, Seoul, Korea; 6 Department of Neurology, Gyengsang national university Changwon Hospital, Changwon, Korea; 7 Department of Critical care medicine & Neurology, Dong-A University Hospital, Busan, Korea; 8 Clinical Trial Center, University of Ulsan College of Medicine and Asan Medical Center, Seoul, Korea; Universitatsklinikum Freiburg, GERMANY

## Abstract

**Background and purpose:**

RAO is caused by various etiologies and subsequent vascular events may be associated with underlying etiologies. Our aim is to investigate the etiologies of RAO, the occurrence of subsequent vascular events and their association in patients with RAO.

**Methods:**

We analyzed data from 151 consecutive patients presenting with acute non-arteritic RAO between 2003 and 2013 in a single tertiary-care hospital. The primary outcome was the occurrence of a vascular event defined as stroke, myocardial infarction, and vascular death within 365 days of the RAO onset. The Kaplan-Meier survival analysis and Cox proportional hazard model were used to estimate the hazard ratio of the vascular events.

**Results:**

Large artery atherosclerosis (LAA) was the etiology more frequently associated with of RAO (41.1%, 62/151). During the one year follow-up, ischemic stroke and vascular events occurred in 8.6% and 9.9% of patients, respectively. Ten vascular events occurred in RAO patients attributed to LAA and 4 occurred in undetermined etiology. RAO patients with LAA had a nearly four times higher risk of vascular events compared to those without LAA (hazard ratio 3.94, 95% confidence interval 1.21–12.81). More than a half of all events occurred within one month and over three fourths of ischemic strokes occurred ipsilateral to the RAO.

**Conclusion:**

After occurrence of RAO, there is a high risk of a subsequent vascular event, particularly ipsilateral stroke, within one month. LAA is an independent factor for the occurrence of a subsequent vascular event. Management for the prevention of secondary vascular events is necessary in patients with RAO especially with LAA. Large clinical trials are needed to confirm these findings.

## Introduction

Retinal artery occlusion (RAO) results in sudden painless visual loss often leaving a visual acuity of counting fingers in case of central RAO. RAO is often associated with critical cerebrovascular and cardiovascular disease that may require systemic treatment.[1, 2]

RAO shares vascular risk factors with stroke and is caused by various etiologies.[3] Ophthalmic artery (OA) stenosis or occlusion can cause retinal ischemic symptoms in patients, and this can be a treatable target vessel with intra-arterial treatment.[4] However, no studies showing etiologies of RAO have investigated the details of OA status through intra-arterial evaluations such as transfemoral cerebral angiography (TFCA). In addition, risk of recurrent vascular events according to their etiologies has not been investigated yet.

Therefore, the aim of this study was to investigate the etiologies of RAO and the occurrence of subsequent vascular events according to their etiologies in the year following a RAO.

## Materials and methods

### Selection of study patients

Between September 2003 and June 2013, 235 consecutive RAO cases were evaluated at single institution and enrolled in RAO registry. RAO was diagnosed with fundoscopy and fluorescein angiography by an ophthalmologist in patients with the sudden onset of monocular partial or complete visual loss. Patients with iatrogenic RAOs, such as those due to accidental intravascular facial filler injection and patients with arteritic RAO or combined retinal vein occlusion were excluded.

RAO was confirmed by an ophthalmologist on the basis of the following criteria: 1) abrupt vision loss with a fundus finding of retinal opacification or cherry red spots and 2) the delayed and incomplete filling of the retinal arteries in fluorescein angiography. The eligibility criteria for intra-arterial thrombolysis (IAT) were symptom duration of ≤24 hours of non-arteritic CRAO (or ≤7 days for incomplete CRAO).[5] Systemic conditions restricting thrombolysis based on current stroke guidelines and iatrogenic cases excluded subjects from receiving IAT. TFCA was carried out in some patients with BRAO by physician’s discretion. After physicians explained a detailed procedure description and potential complications and obtained the informed consent to subjects, TFCA or IAT was performed.

As diagnosed by an ophthalmologist, patients with RAO were registered prospectively in the RAO database. Concurrently, all of patients with RAO received stroke evaluation and management by the neurology department. Principle management including the administration of antithrombotics and statin based on stroke etiologies was in accordance with current stroke guidelines.

### Outcome measurement and definitions

RAO registry database included patient demographics, vascular risk factors and etiological stroke classification, diagnostic work-up, in-hospital management including acute treatment, laboratory values, magnetic resonance imaging (MRI) and magnetic resonance angiography (MRA) of the brain, and clinical events occurring after RAO. Ischemic heart disease was defined as a composite variable including myocardial infarction, unstable angina, percutaneous coronary intervention, coronary artery bypass grafting, and percutaneous transluminal coronary angiography. All of these data were collected prospectively to monitor and improve the quality of stroke care and our study was approved by the local institutional review board of Seoul National University Bundang Hospital, Republic of Korea. (Institutional Review Board approval No. B- 1510/318-103).

With regard to the etiology of RAO, we evaluated patients for a significant steno-occlusive lesion in the extracranial carotid artery, intracranial carotid artery, and OA through elective TFCA including IAT. We also performed transthoracic and transesophageal echocardiography, 24-hour Holter monitoring, MRI, and MRA. Clinical events were evaluated by board-certified experts in neurology at the outpatient clinic or through a structured telephone interview by a trained nurse at one year post RAO.

The etiological subtype of RAO was based on medical and radiological data and was assessed by stroke physicians according to the Trial of ORG 10172 in Acute Stroke Treatment (TOAST) classification with some modifications.[6] The following original subgroups were included in the analysis; large artery atherosclerosis (LAA) including the internal carotid artery and OA; cardioembolism; other determined etiology; undetermined etiology including two or more and negative (UD-negative) etiology, and incomplete evaluation. Based on TOAST classification, stroke etiology was dichotomized into the two following categories: RAO with LAA vs. RAO without LAA.

Primary outcomes were defined as a vascular event including any kind of stroke, myocardial infarction, and vascular death within 365 days of the RAO occurrence. Stroke was defined as a sudden focal neurologic deficit with a consistent cerebral ischemic or hemorrhagic lesion and included transient ischemic attacks.

### Statistical analysis

Bivariate analysis was performed using Pearson’s χ^2^-test or Fisher’s exact test for categorical variables and Student’s t-test or the Mann-Whitney U test for continuous variables as appropriate. We divided our patients into two groups according whether they received TFCA or IAT (TFCA group), or not (MRA group), for the evaluation of the etiological subtypes of RAO. The Kaplan-Meier survival analysis was performed to estimate event rate. Independent associations of vascular events with patients’ demographic and etiological factors were determined using the Cox proportional hazard model with adjustment. Adjustments were made for the predefined variables (age and sex) and potential confounders whose *P* values were < 0.2 in the comparison of the two groups according to a vascular event in the Cox proportional hazard model. A two-tailed *p <* 0.05 was considered significant. All statistical analyses were performed using the SAS version 9.3 (SAS Institute Inc, Cary, NC, USA).

Additionally, the Cox proportional hazard model was used to estimate survival rates after RAO according to stroke etiology (RAO with LAA vs. RAO without LAA). We investigated the duration from the RAO occurrence to the vascular event occurrence for evaluating the time-dependent incidence using the following three time periods: days 1–30, days 31–90, and days 91–365.

## Results

Over the 10 years, 200 patients with RAO were evaluated. Among them, 49 patients refused admission and detailed evaluations for risk factors and etiology of RAO were not available. Finally 151 consecutive patients with RAO were enrolled in this study (S1 Fig). RAO patients included 119 (78.8%) central RAOs and 32 (21.2%) branch RAOs with a mean age of 60.8 y (range, 14–92 y). TFCA or IAT was performed in 80 RAO patients. In terms of vascular risk factors, 87 of the 151 patients (57.6%) had hypertension, 35 (23.2%) had diabetes, and 35 (23.2%) had hyperlipidemia. Sixteen patients (10.6%) already had stroke and transient ischemic attack (TIA) before RAO occurrence. Atrial fibrillation or valvular heart disease was present in only 8 patients (6%) (Table 1).

**Table 1:** Demographics of retinal artery occlusion.

	Evaluation of RAO (n = 151)
**Age, mean**	60.8 ± 15.3
**Sex (male)**	102 (67.5%)
**Side of RAO (right)**	75 (49.7%)
**Type of RAO**	
** Central RAO**	119 (78.8%)
** Branch RAO**	32 (21.2%)
**Risk factors**	
** History of stroke or TIA**	16 (10.6%)
** Hypertension**	87 (57.6%)
** Diabetes mellitus**	35 (23.2%)
** Hyperlipidemia**	35 (23.2%)
** Ischemic heart disease**	17 (11.3%)
** Valvular heart disease or Atrial fibrillation**	8 (6.0%)

RAO, retinal artery occlusion; TIA, transient ischemic attack

With regard to etiology, 62 patients (41.1%) had LAA, the most common etiological factor. About 40% of our patients had a RAO of undetermined etiology. There was a difference in the proportions of TOAST classification and the type of RAO between the TFCA group and the MRA group. In the TFCA group, the primary etiology was LAA in 48.8% (39/80) of patients, a steno-occlusive lesion of the OA was present in 20% (16/80) of patients, and occlusive disease limited to the OA was present in 8.8% (7/80) of patients. On the other hand, undetermined etiology was reported as a main etiology in the MRA group (Table 2 and S1 Table).

**Table 2:** Etiological subtypes of retinal artery occlusion.

Etiologic subtypes	Evaluation of RAO (n = 151)	TFCA group (n = 80)	MRA group (n = 71)
Large artery atherosclerosis	62 (41.1%)	39 (48.8%)	23 (32.4%)
Carotid artery	_	23 (28.8%)	_
Ophthalmic artery	_	7 (8.8%)	_
Combined ophthalmic and carotid artery	_	9 (11.3%)	_
Cardioembolism	6 (4.0%)	5 (6.3%)	1 (1.4%)
Other determined	5 (3.3%)	1 (1.3%)	4 (5.6%)
Undetermined	61 (40.4)	27 (33.8%)	34 (47.9%)
Two or more	3 (2.0%)	2 (2.5%)	1 (1.4%)
Negative	58 (38.4%)	25 (31.3%)	33 (46.5%)
Incomplete	17 (11.3%)	8 (10.0%)	9 (12.7%)

RAO, retinal artery occlusion; TFCA, transfemoral cerebral angiography

The TFCA group was defined as subjects who received TFCA or intra-arterial thrombolysis.

**Fig 1:**
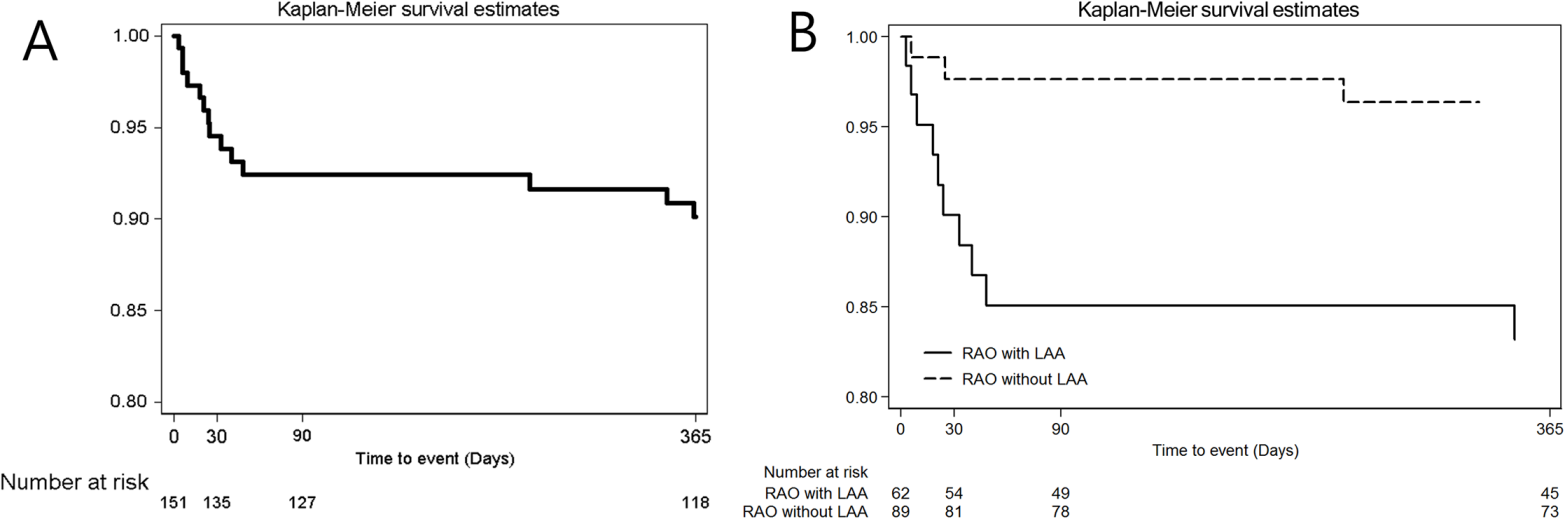
Vascular events in patients with retinal artery occlusion. Kaplan-Meier curves of vascular event according to period in overall subjects; days 1–30, days 31–90, and days 91–365 (A) and according to etiologies; RAO with LAA vs RAO without LAA (B) within 1 year after retinal artery occlusion occurrence. RAO, retinal artery occlusion; LAA, large artery atherosclerosis.

After occurrence of RAO, 18 of 151 (11.9%) patients were lost to follow-up within one year. One-year rate of occurrence for subsequent vascular events was 9.9% (14 events). Ischemic stroke occurred in 13 patients (1-year rate, 8.6%) and no patients experienced hemorrhagic stroke. Non-vascular death occurred in one patient (0.7%), which was censored for the analysis. Over three fourths of ischemic strokes occurred ipsilateral to the RAO. Details of the vascular events occurring during the one year follow-up are presented in Table 3. The rate of vascular events was nearly four times higher in RAO patients with LAA (hazard ratio 3.94, 95% confidence interval 1.21–12.81) than in RAO patients without LAA (Fig 1). In the Cox proportional hazard model for predicting vascular events, LAA was the only independent risk factor for vascular event (Table 4). Ten vascular events occurred in RAO patients attributed to LAA and 4 occurred in undetermined etiology. No events occurred in the cardioembolism, other determined and incomplete etiologies. With regard to vascular event according to period after RAO occurrence, 8 (57.1%) events occurred within 30 d of the RAO, three (21.4%) occurred between 30 and 90 d, and three (21.4%) occurred between 91 and 365 d.

**Table 3:** One-year clinical event rate.

	One-year event rate
Vascular events	9.9%
Hemorrhagic stroke	-
Ischemic stroke^a^	8.6%
Ipsilesional^a^	6.6%
Contralesional	2.0%
Myocardial infarction	0.7%
Vascular death	-
Non-vascular death	0.7%

^a^ This figure includes one patient with transient ischemic attack.

**Table 4:** Independent variables associated with vascular events by the Cox proportional hazard model.

	Crude analysis				Adjusted analysis			
	HR	95% CI		P-value	HR	95% CI		P-value
Age	1.00	(0.96	1.03)	0.81	0.99	(0.96	1.02)	0.49
Sex (male)	0.88	(0.30	2.63)	0.82	0.81	(0.27	2.48)	0.71
Side of RAO (right)	1.81	(0.61	5.41)	0.29				
Type of RAO								
Central RAO	3.79	(0.50	28.9)	0.20	3.45	(0.45	26.46)	0.23
Branch RAO	Reference				Reference			
Risk factors								
History of stroke or TIA	0.65	(0.09	5.00)	0.68				
Hypertension	1.27	(0.43	3.80)	0.67				
Diabetes mellitus	1.34	(0.42	4.26)	0.62				
Hyperlipidemia	0.55	(0.12	2.46)	0.44				
Valvular heart disease or atrial fibrillation	0.55	(0.03	10.14)	0.69				
Etiologic subtypes								
Large artery atherosclerosis	3.78	(1.18	12.05)	0.02	3.94	(1.21	12.81)	0.02
Others	Reference				Reference			

HR, hazard ratio; CI, confidence interval; RAO, retinal artery occlusion; TIA, transient ischemic attack

## Discussion

This study demonstrated that LAA was more frequently associated with RAO than other etiologies and RAO patients with LAA had a nearly four times higher risk of vascular events compared to those without LAA.

The positive association between retinal stroke or emboli and a subsequent vascular event has been presented in prior studies.[7–10] In our study, the rate of vascular events and strokes in the first year following a RAO was 9.9% and 8.6%, respectively. Our results are similar to those of previous studies reporting a range of 7.8–26%, although these prior results were based on heterogeneous study populations.[7, 9–12] This result was also similar to those of studies using an ischemic stroke cohort.[13]

RAO patients attributed to LAA had about a four times higher risk for the occurrence of vascular events during the one year follow-up after RAO than those with other etiologies. There has been no study focused on recurrent risk of subsequent vascular event according to RAO etiologies. However, Helenius et al. showed that approximately one out of every four patients with monocular visual loss caused by ischemia in the retinal circulation had a concurrent acute brain infarct with diffusion-weighted imaging and their probability was higher in patients with an identified etiology than those with an undetermined one. Major subtype of identified etiology was LAA. [14] Lovett et al. reported risk of recurrent stroke by etiologic subtype after ischemic stroke occurrence and clearly demonstrated that the rate of recurrent stroke was also highest in ischemic stroke patients attributed to LAA.[15]

Over half of subsequent vascular events occurred within one month of the RAO event. Moreover, in most patients the ischemic stroke occurred ipsilateral to the RAO. Previous studies have reported that the incidence of experiencing a subsequent stroke was highest in the first month after retinal ischemia.[7, 11, 12, 16] Considering these findings, early treatment and appropriate interventions, such as carotid endarterectomy or carotid stenting, may be carefully considered for the prevention of a second vascular event.

Our study provided data regarding the incidence of steno-occlusion of the OA, in addition to carotid artery disease, using a protocol based on TFCA. The main etiology presented a contrast between two groups according to receiving TFCA or not: LAA in the TFCA group vs. UD-negative in the MRA group. Sixteen patients (20%) of TFCA group had steno-occlusion of the OA and seven patients (8.8%) had pure steno-occlusion of OA, without evidence of carotid artery atherosclerosis. Babikian et al. reported that the most common identifiable etiology (22.1%) of RAO in American subjects was stenosis of the extracranial carotid artery and OA stenosis was reported in only 2.6%.[11] However, Asians have a higher incidence of intracranial atherosclerosis than Caucasians. Anatomically, the retinal artery arises from the OA, the first intracranial branch of the internal carotid artery. Our study implies that steno-occlusive lesions of the OA are an important, but less often recognized etiology, especially in Asian RAO patients. Several studies have reported pure steno-occlusion at the origin of the OA, with no evidence of carotid stenosis, in patients with transient mono-ocular blindness.[4, 17, 18] The OA may be a potential therapeutic target vessel in patients with an abrupt decline of vision from reduced retinal perfusion due to steno-occlusion of the OA.[4] However, adverse reactions related with endovascular treatment of intracranial arteries should be carefully considered in clinical decision making.

Systemic vascular risk factors for RAO overlapped with those for ischemic stroke. Hypertension was the most common vascular risk factor in RAO patients followed by diabetes mellitus and hyperlipidemia. In previous studies, the vascular risk factors that predispose patients to atherosclerotic disease were present in a larger proportion of patients with RAO than that seen in the general population.[8, 11, 19] Cardioembolism as an etiology, such as atrial fibrillation and valvular heart disease, was present in 6% of RAO patients. Leisser et al. reported about 4.3% of patients with vegetations on the cardiac valves and 10.3% of patients with other reasons for possible cardioembolism diagnosed with TEE. Atrial fibrillation was 9.4%.[20] These finding was similar to our results.

Major vascular risk factors for RAO are modifiable and can predispose patients to atherosclerotic disease. Most of subsequent vascular events occurred within 1 month after the RAO occurrence. However, only one third of ophthalmologists will immediately evaluate patients with an acute RAO; most ophthalmologists send patients to the emergency room for inclusion in clinical trials.[21] Although we utilized a clinical pathway for immediate evaluation in patients with RAO, about one in four RAO patients (49/200) refused any evaluation for risk factors and etiology, and 11.9% were lost to follow-up at one year. An effort to raise awareness of RAO among patients, as well as physicians, is necessary.

This study had several limitations. First, only 151 patients were included, which is not a sufficient number for a statistically significant evaluation. However, many patients underwent a more detailed evaluation of the OA by TFCA, allowing a comparison of the two groups regarding etiology. Second, TOAST classification based on stroke etiology has limitation about causal relationship. Leisser et al. reported about a probability of 12.6% or lower for clots to be washed into the OA, which means that at least 87.4% of clots in the carotid artery are washed into the brain.[22] Based on this finding, it is unclear whether RAO is caused by LAA or other reasons in even RAO patients with LAA. Nevertheless, it is definite that LAA is a common comorbidity in RAO patients. Third, our patient population was Asian; as such, our results should be interpreted with caution. These finding need to be validated among other ethnic cohorts before our findings are generalizable. Finally, many patients with RAO were lost to follow-up at one year, possibly because of a lack of insight into the disease by both patients and physicians.

Because RAO patents have a high risk of a subsequent vascular event, an evaluation for risk factors and the etiology of the RAO should be performed. LAA is a major etiology for the occurrence of subsequent vascular events and prompt aggressive management should be instituted to decrease the risk of a subsequent vascular event. Further investigations of the etiologies and vascular events associated with RAO in larger ethnically diverse populations are necessary.

## Supporting information

S1 Fig Demographics and etiologic subtypes of retinal artery occlusion by intra-arterial evaluation. (DOCX)

S1 Table Flow chart of enrolled subjects with non-arteritic RAO. (DOCX)
