# Supplementary material for: Retinal artery occlusion and associated recurrent vascular risk with underlying etiologies
Source: PLoS One. 2017 Jun 1;12(6):e0177663. doi: 10.1371/journal.pone.0177663 (PMC5453434; doi:10.1371/journal.pone.0177663)
Supplement: S1 Fig — (DOCX) [file pone.0177663.s001.docx]

**S1 Fig. Flow chart of enrolled subjects with non-arteritic RAO.**

**
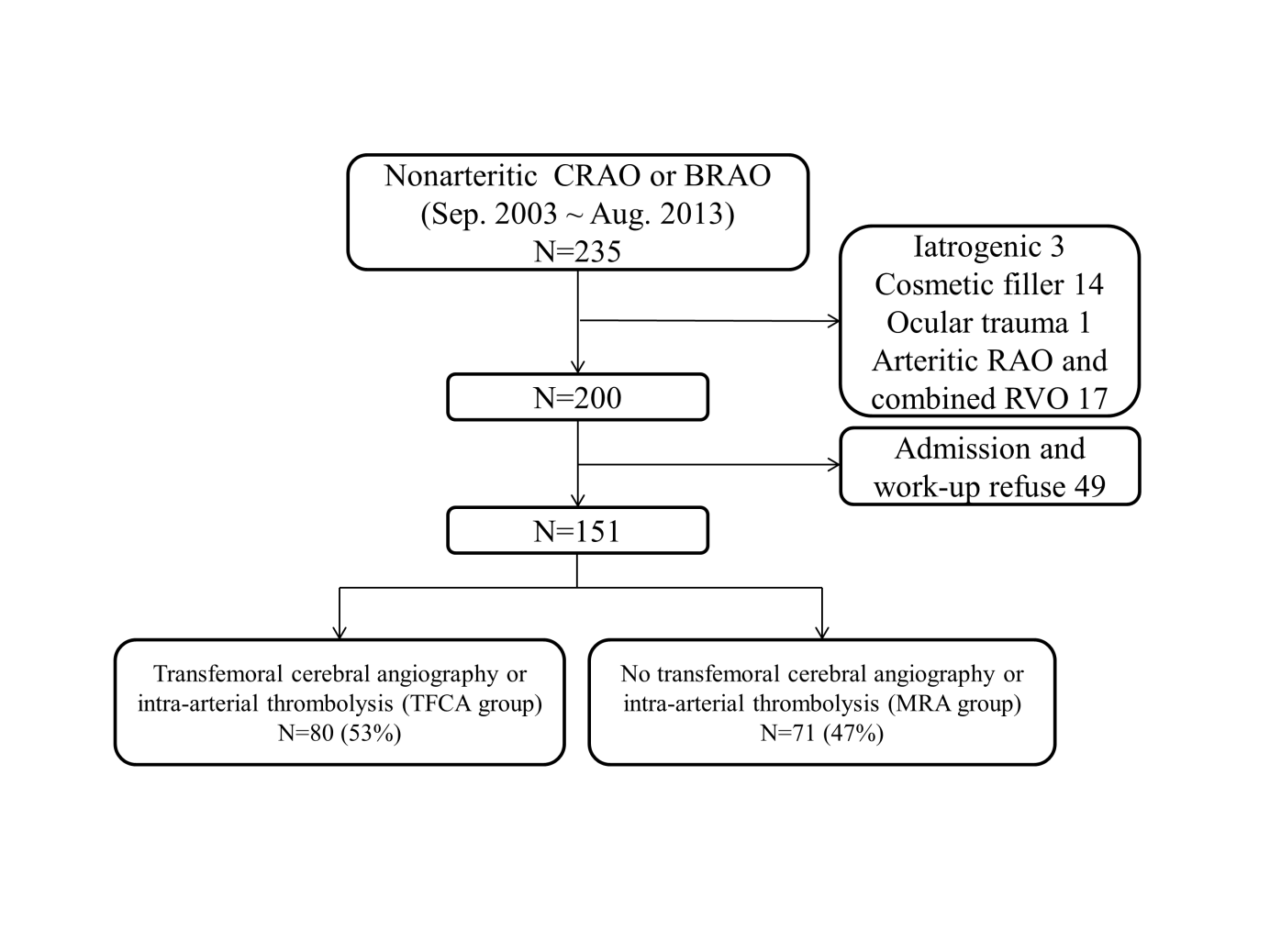
**

RAO, retinal artery occlusion; CRAO, central RAO; BRAO, branch RAO; RVO, retinal vein occlusion; TFCA, transfemoral cerebral angiography
