# Supplementary material for: Retinal artery occlusion and associated recurrent vascular risk with underlying etiologies
Source: PLoS One. 2017 Jun 1;12(6):e0177663. doi: 10.1371/journal.pone.0177663 (PMC5453434; doi:10.1371/journal.pone.0177663)
Supplement: S1 Table — (DOCX) [file pone.0177663.s002.docx]

**S1 Table. Demographics and etiologic subtypes of retinal artery occlusion by intra-arterial evaluation.**

|  | TFCA group  N=80 | MRA group  N=71 |
| --- | --- | --- |
| Age, mean | 60.4 ± 14.3 | 61.2 ± 16.5 |
| Sex (male) | 55 (66.2%) | 47 (66.2%) |
| Side of RAO (right) | 37 (46.3%) | 39 (54.9%) |
| Type of RAO |  |  |
| Central RAO  Branch RAO | 72 (90.0%)  8 (10.0%) | 49 (66.2%)  24 (33.8%) |
| Risk factors |  |  |
| History of stroke or TIA  Hypertension  Diabetes mellitus  Hyperlipidemia  Ischemic heart disease  Valvular heart disease or Atrial fibrillation | 7 (8.8%)  47 (58.8%)  21 (26.3%)  20 (25.0%)  10 (12.5%)  7 (8.8%) | 9 (12.7%)  40 (56.3%)  14 (19.7%)  15 (21.1%)  7 (9.9%)  2 (2.8%) |

RAO, retinal artery occlusion; TIA, transient ischemic attack; TFCA, transfemoral cerebral angiography

TFCA group was defined as subjects who received TFCA or intra-arterial thrombolysis
